# Value of CT‐guided Core Needle Biopsy in Diagnosing Spinal Lesions: A Comparison Study

**DOI:** 10.1111/os.12418

**Published:** 2019-02-14

**Authors:** Yun Liang, Peng Liu, Li‐bo Jiang, Hou‐lei Wang, An‐nan Hu, Xiao‐gang Zhou, Xi‐lei Li, Hong Lin, Dong Wu, Jian Dong

**Affiliations:** ^1^ Department of Orthopaedics, Shanghai Zhongshan Hospital Fudan University Shanghai China; ^2^ Department of Radiology, Shanghai Zhongshan Hospital Fudan University Shanghai China

**Keywords:** C‐arm fluoroscopy guidance, CT guidance, Core needle biopsy, Diagnostic accuracy rate, Spinal lesion

## Abstract

**Objective:**

A retrospective study was designed to evaluate the effectiveness of CT‐guided core needle biopsy in diagnosing spinal lesions through comparison with C‐arm guidance.

**Methods:**

From April 2013 to July 2017, a total of 188 patients, who suffered from spinal lesions or had malignant tumor history with a new spinal fracture, were included in this study. There were 96 men and 92 women, with an average of 57.1 years. A total of 238 core needle biopsies were performed. A total of 140 core needle biopsies were carried out under C‐arm guidance in 102 patients (group 1); 98 core needle biopsies were carried out under CT guidance in 86 patients (group 2); 108 core needle biopsies were performed in thoracic vertebrae, 116 were in lumbar vertebrae, and 14 were in sacral vertebrae. Seventy‐eight patients accepted surgical treatment after biopsies. For these patients, the histological pathologies of the biopsy and surgery were compared to evaluate the accuracy of the biopsy. For the other 110 patients who did not receive surgical treatment, the treatment response and the clinical course were used to evaluate the accuracy of the biopsy. The success rate, the diagnostic accuracy rate, the true positive/negative rate, and complications of the two groups were calculated and compared.

**Results:**

There were no significant differences in sex, age, and lesion sites between the C‐arm guidance group (group 1) and the CT guidance group (group 2). There were no complications in the two groups. Pathological diagnoses were established in 232 of 238 biopsies. They revealed that 52 were primary malignant tumors, 12 were benign tumors, 70 were metastatic tumors, 4 were tuberculosis, and 94 were classified as “other.” The success rate of group 2 was higher than that of group 1, but it was not statistically significant (95.7% *vs* 100%; *P* = 0.098). According to the final diagnosis, the diagnostic accuracy rates were calculated and compared. There was no significant difference between the two groups (95.5% *vs* 96.9%; *P* = 0.835). The kappa coefficient was used to analyze the concordance between the histological pathologies of the biopsy and the final diagnosis in the two groups. The kappa values of the two group were 0.909 and 0.939, respectively. The results showed good consistency in both groups, but seemed better for group 2.

**Conclusion:**

CT‐guided core needle biopsy is a relatively safe and effective procedure for diagnosing spinal lesions with a high diagnostic accuracy rate and few complications.

## Introduction

Because of the need for precision in medical care, an accurate biopsy is necessary to make an accurate diagnosis for spinal lesions. Although modern imaging technology is well developed, a precise histological diagnosis is essential for further treatment. Traditionally, incisional biopsy was considered the gold standard for pathological evaluation. However, Mankin *et al.* report a complication rate of 15.9% for incisional biopsy, including hematoma, infection, and tumor contamination, affecting approximately 8% of treatment plans^1^. Meanwhile, for deep lesions, such as spinal lesions, an open incisional biopsy is difficult to perform and there is significant risk of complications^2^.

Percutaneous core needle biopsy is suitable for spinal lesions^3^. The procedure has a low complication rate and a relatively low cost with high accuracy. Some surgeons report that core needle biopsy can be performed under C‐arm fluoroscopy guidance for spinal lesions, and has good outcomes^4^. However, the images are only two‐dimensional, and they are not clear enough when soft tissue is involved. As a result, some specimens cannot be obtained correctly using C‐arm‐guided core needle biopsies due to their position in vertebral body or soft tissues.

Recently, when core needle biopsies were performed under CT guidance, they were demonstrated to be more accurate and safer for patients than C‐arm‐guided biopsies. CT guidance could show the lesions more clearly and avoid vascular and nerve injury, especially when the lesions were in deep sites, such as in the vertebral body^5^, ^6^, ^7^.

CT‐guided core needle biopsy is an advantageous technique for diagnosing spinal lesions; however, it is not widely used in hospitals and is unfamiliar to some physicians. The purpose of our study is to retrospectively evaluate the effectiveness of CT‐guided core needle biopsy in the diagnosis of spinal lesions by comparing the diagnostic accuracy between CT‐guided core needle biopsy and C‐arm‐guided core needle biopsy.

## Methods

### *Patient Demographics*

From April 2013 to July 2017, a total of 188 patients with spinal lesions or a history of malignant tumors who developed a new spinal fracture were included in this study. The inclusion criteria for selection of patients were: (i) patients with a spinal lesion for which it was difficult to clarify the diagnosis; (ii) patients who had a history of malignant tumor who had developed a spinal lesion highly suggestive of metastasis; and (iii) elderly patients with a vertebral compression fracture for which the imaging could not identify osteoporosis or a tumor. The only absolute exclusion criteria for biopsy was a severe bleeding tendency. Therefore, bleeding and clotting function were tested before biopsy in every patient. There were 96 men and 92 women, with an average age of 57.1 years (range, 24–83 years). A total of 238 core needle biopsies were performed in 188 patients. From April 2013 to September 2015, core needle biopsy was performed 140 times in 102 patients under C‐arm guidance (group 1). Then, from September 2015 to July 2017, core needle biopsy was performed 98 times in 86 patients under CT guidance (group 2). A total of 108 biopsies were in thoracic vertebrae, 116 were in lumbar vertebrae, and 14 were in sacral vertebrae (Table 1).

**Table 1:** Patient demographics data

Parameter	Group 1	Group 2	Total	*P*‐value
Number of cases	102	86	188	
Ages (mean ± SD, years)	58.6 ± 12.6	55.3 ± 12.9	57.1 ± 12.9	0.075
Gender (M/F)	50/52	46/40	96/92	0.541
Lesions site	140	98	238	0.492
Thoracic vertebrae	68	40	108	
Lumbar vertebrae	64	52	116	
Sacral vertebrae	8	6	14	

F, female; M, male.

There were no statistically significant differences between the two groups in terms of sex distribution, age, and lesion sites. Preoperative examination, including physical and radiological imaging, histological pathologies of the biopsies, subsequent therapy (i.e. surgery, radiotherapy and/or chemotherapy, anti‐infective therapy or antituberculosis therapy), and clinical course were recorded. Seventy‐eight patients accepted surgical treatment after biopsies (e.g. percutaneous vertebroplasty [PVP], palliative decompression surgery, and total en‐bloc spondylectomy). The other 110 patients did not receive surgical treatment. All patients provided informed consent before the biopsy.

### *Biopsy Procedure*

#### *CT‐guided*

**Figure 1:**
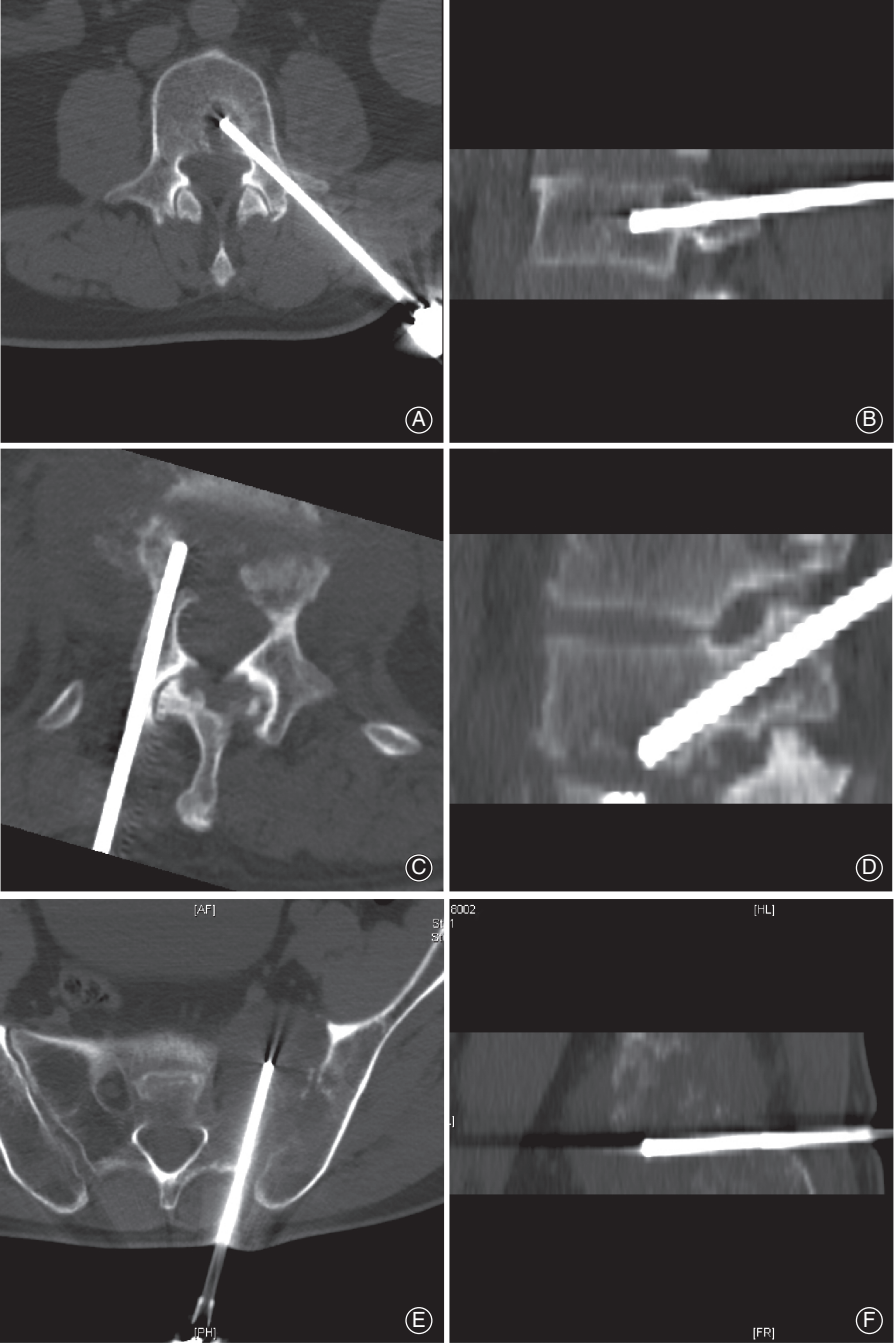
Images of CT‐guided biopsy. (A, B) The lesion is located in the posterior part of the vertebral body, close to the spinal canal. Biopsy confirmed metastatic low differentiation adenocarcinoma. (C, D) Transpedicular approach to an L_1_ lesion, located close to the inferior endplate. Biopsy showed a plasmacytosis, but a diagnosis of tuberculosis was eventually given by a multidisciplinary team. (E, F) Biopsy was performed in the left sacroiliac joint and cytology was compatible with diffuse large B‐cell lymphoma.

All the CT‐guided core needle biopsies were performed by a senior orthopaedic surgeon with the assistance of a radiologist. After placing the patient in a prone position on the CT table, a CT scan was taken to locate the position of the lesion. According to the images of the CT scan images, the best puncture point and puncture path were determined. Before local anesthesia with 1% lidocaine, the skin was sterilized and draped. Then, the needle with trocar was inserted through the predetermined puncture point and along the predetermined puncture path. CT scans were applied to correct the direction of the needle: to avoid nerve roots, the spinal canal, vessels, and the other major organs; and to confirm the needle at the position of the lesion (Fig. 1). The specimens were slowly withdrawn from the puncture path. The wound was covered with compressive dressing. The specimen was immediately fixed in formalin, and then sent to the pathology department.

#### *C‐Arm‐guided*

Unlike CT‐guided core needle biopsies, C‐arm‐guided core needle biopsies are monitored by fluoroscopy only. The C‐arm‐guided core needle biopsies were performed by a senior orthopaedic surgeon without a radiologist. The remainder of the biopsy steps are similar to those of the CT‐guided core needle biopsy.

### *Assessment Index*

Histological pathologies of the biopsy and surgery were compared to evaluate the accuracy of biopsy in 78 patients who accepted surgical treatment. According to Rimondi's research^8^, for the other 110 patients who received a non‐surgical treatment, the treatment response and the clinical course were used to evaluate the accuracy of the biopsy. The success rate of the biopsy, the diagnostic accuracy rate, the true positive rate, the true negative rate, and the complication rate of the two biopsy method groups were calculated. The success of the biopsy was defined as that the specimen could be obtained and a pathological diagnosis could be made, whether correct or not. The diagnostic accuracy was defined as that the biopsy pathological diagnosis was the same as the final diagnosis.

### *Statistical Analysis*

Data analysis was performed using the SPSS 21.0 statistics software program (SPSS, Chicago, USA). The difference in sex distribution, age, and puncture sites between the two groups were compared using the χ^2^‐test. The χ^2^‐test was also used to compare the success rate of biopsy and the diagnostic accuracy rate between the two groups. The kappa coefficient was used to evaluate concordance between the histological pathologies of the biopsy and the final diagnosis in the two groups. *P* < 0.05 was considered statistically significant.

## Results

### *Complications and Pathological Diagnoses of Biopsies*

There were no complications in all core needle biopsies of 188 patients in either groups 1 or 2. The pathological diagnoses of 232 biopsies were confirmed. Of these 232 specimens, 52 (22.4%) were primary malignant tumors, 12 (5.2%) were benign tumors, 70 (30.2%) were metastatic tumors, 4 (1.7%) were tuberculosis, and 94 (40.5%) were classified as “others” (including nonspecific infection, chronic inflammation, and fracture). The remaining 6 biopsies failed to establish a clear pathological diagnosis due to the specimens containing inadequate tissue.

### *Success Rate and Diagnostic Accuracy Rate in the Two Groups*

The total success rate of biopsy was 97.5% (232/238): 95.7% (134/140) in group 1 and 100% (98/98) in group 2 (Table 2). However, the difference between the two groups was not statistically significant (*P* = 0.098). According to the final diagnosis, the total diagnostic accuracy rate of biopsies was 96.1% (223/232): 95.5% (128/134) in group 1 and 96.9% (95/98) in group 2 (Table 2). There were 6 biopsies with a misdiagnosis in group 1, and 3 in group 2 (Table 3). Although the diagnostic accuracy rate was higher in group 2, there was no statistically significant difference between the two groups in the diagnostic accuracy rate (*P* = 0.835). The true positive rate and the true negative rate of diagnosing a spinal malignant tumor were, respectively, 97.3% and 93.2% for C‐arm‐guided biopsy, and 96.2% and 97.8% for CT‐guided biopsy.

**Table 2:** Comparison of two biopsy methods

Parameter	Group 1	Group 2	Total	*P*‐value
Success rate (%)	95.7	100	97.5	0.098
Diagnostic accuracy rate (%)	95.5	96.9	96.1	0.835
True positive rate (%)^†^	97.3	96.2		
True negative rate (%)^‡^	93.2	97.8		
kappa value^§^	0.909	0.939		

†, ‡ The true positive rate and the true negative rate of diagnosing spinal malignant tumor.

§ The concordance between the histological pathologies of the biopsy and the final diagnosis.

**Table 3:** Summary of cases of misdiagnosis

Group	Gender/Age (years)	Site	Diagnosis by biopsy	Final diagnosis
1	M/61	L_1_	No evidence of tumor	Metastatic tumor
1	F/54	L_5_	No evidence of tumor	Metastatic tumor
1	M/38	L_4_	Chronic inflammation	Metastatic tumor
1	M/44	T_9_	Chronic inflammation	Tuberculosis
1	F/50	T_4_	Chronic inflammation	Tuberculosis
1	M/46	L_5_	Hematoma	Tuberculosis
2	F/55	T_12_	No evidence of tumor	Plasmacytoma
2	M/64	L_1_	Plasmacytosis	Tuberculosis
2	F/57	L_4_	No evidence of tumor	Metastatic tumor

M, male; F, female

Using the kappa test, we analyzed the concordance between the histological pathologies of the biopsy and the final diagnosis in the two groups. The kappa values of the two groups were 0.909 and 0.939, respectively. The results showed good consistency in both groups but seemed better for group 2.

## Discussion

Accurate diagnosis of spine lesions is important for their further management. There is no doubt that advances in modern imaging technology have improved the sensitivity of diagnosing and differentiating spinal lesions. However, a definitive diagnosis is always based on histological evidence. Therefore, a biopsy is necessary for diagnosis and to provide the appropriate treatment^6^. Conventionally, incisional biopsy harvested sufficient specimens and provided a high diagnostic success rate for the disease. However, for some deep structures, especially in the spine, incisional biopsy is often associated with a high risk of complications, such as hematoma, infection, and tumor contamination^1^. Thus, percutaneous core needle biopsy under CT guidance was developed.

**Figure 2:**
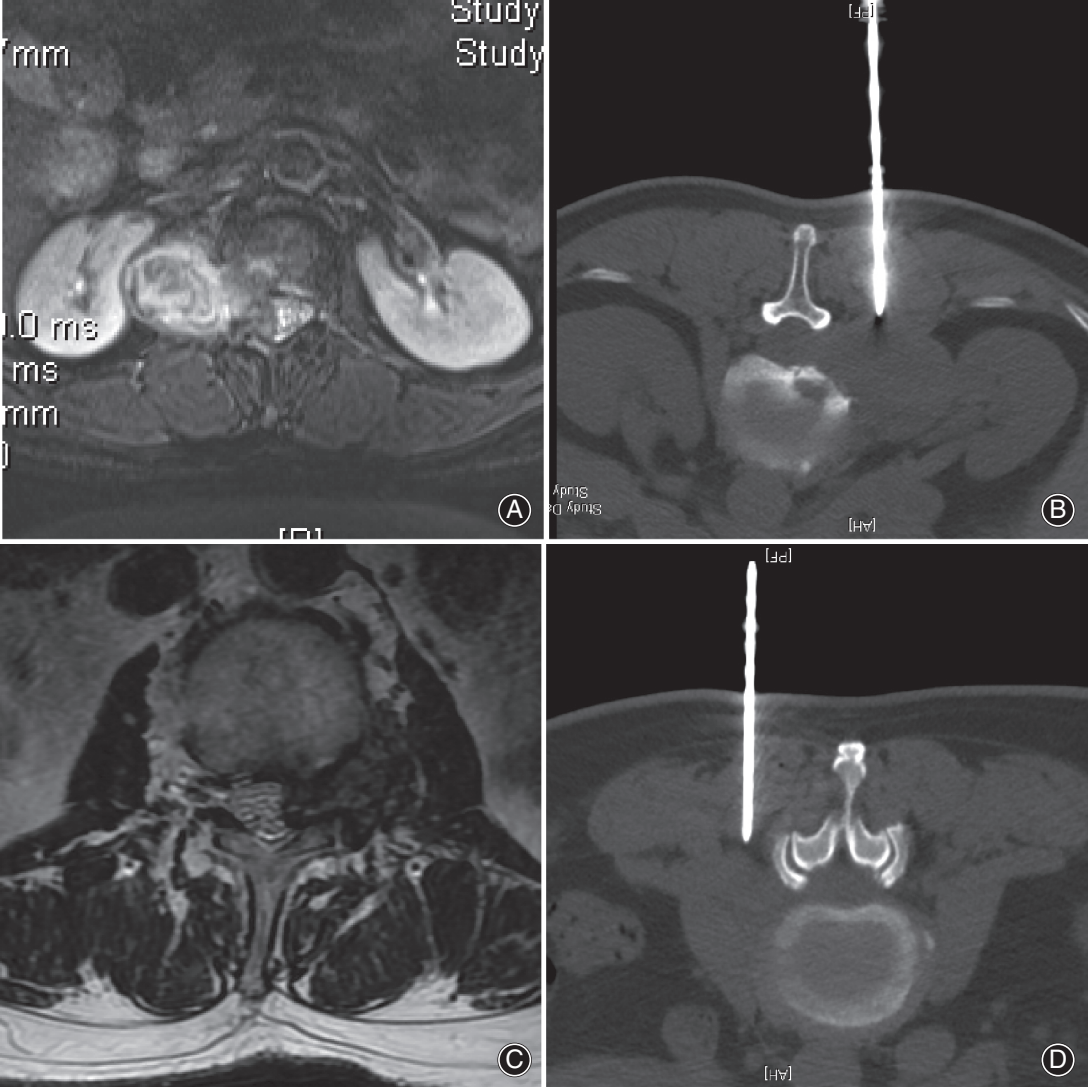
(A) MRI showed a paravertebral soft tumor in a 61‐year‐old woman. (B) A diagnosis of schwannoma was given by CT‐guided biopsy. (C) MRI showed a soft tumor located in the intervertebral foramina in a 76‐year‐old man where it was difficult to guide by C‐arm only. (D) CT‐guided biopsy revealed it was a metastasis of renal cell carcinoma.

CT scans can clearly show the structure of the spine and adjacent blood vessels and nerves. The best puncture point and puncture path can be determined to avoid damage to important structures. At the same time, the puncture needle can be accurately positioned in the targeted area under CT guidance. In this study, the success rate of biopsy was 100% in the CT‐guided biopsy group, which was higher than that in the C‐arm‐guided biopsy group (95.7%). However, the difference was not statistically significant (*P* = 0.098). The higher success rate of group 2 could be attributed to several factors. The C‐arm fluoroscopy provides 2D images, which do not clearly show the lesions. In addition, soft tissue tumors cannot be visualized clearly in fluoroscopy. Those shortcomings may lead to missing the needed biopsy specimen. The CT scan can overcome these disadvantages (Fig. 2). However, the difference between the two groups was not statistically significant (*P* = 0.098).

In our study, the total diagnostic accuracy rate was 96.1%: 95.5% (128/134) for group 1 and 96.9% (95/98) for group 2. The accuracy rate for the CT‐guided group was higher, but there was not statistically significant difference. It appears that C‐arm‐guided biopsy has a similar success rate and diagnostic accuracy to CT‐guided biopsy in spinal lesions. However, before September 2015 in our hospital, when a lesion was located at a special position in vertebrae or a soft‐tissue mass, which was not expected to be successfully harvested under C‐arm guidance, the biopsy would not be performed, or an incisional biopsy was performed. After September 2015, all biopsies were performed under CT guidance. This result might have a little bias, which was a limitation of this study. However, it is difficult to find a patient who will accept both biopsy methods, and there were no statistically significant differences between the two groups in term of sex distribution, age, and puncture sites. Therefore, the results were still reliable.

There were four misdiagnosed biopsies that had final diagnoses of tuberculosis (Table 3). Thus, we found a low diagnostic accuracy rate in diagnosing tuberculosis by biopsy. However, some patients who were initially suspected of having tuberculosis by imaging examinations had received anti‐tuberculosis therapy before biopsy. As a result, the lesions may have alleviated and improved. In addition, the typical specimen may not be harvested at the time of biopsy, which may affect the accuracy of diagnosis. Consistently, a significantly low diagnostic accuracy rate was seen in the infection cases, which the author considered could be attributed to those cases that received antibiotics before biopsy^9^. Therefore, for these patients, we need to make a diagnosis by synthesizing all information, including clinical course, imaging examinations, and biopsy result.

The diagnostic accuracy rate of CT‐guided core needle biopsy in spinal lesions reported in previous published studes varies from 71.0% to 97.3%^4^, ^6^, ^8^, ^9^, ^10^, ^11^. Kornblum *et al*. report a diagnostic accuracy rate of 71% in 1998, which is lower than that of our study and others^10^. However, that was over 20 years ago. Presently, the CT image has a higher resolution with 3D reconstruction, and the biopsy instrument is more advanced than 20 years ago. Our result (96.9%) was comparable to those from recent published studies.

Many factors affect the diagnostic accuracy of core needle biopsy in spinal lesions, including the experience of the physician who is performing the biopsy and pathological examination, the lesion size, the lesion location, and the lesion nature^12^. To increase diagnostic accuracy, we believe that the following points should be taken into consideration. First, before core needle biopsy, clinical manifestations, image examination, and treatment process should be analyzed thoroughly to learn the relation between lesions and the spinal cord, as well as the great vessels. Second, the puncture path should always be designed based on the most significant part of the lesion to draw the actual contents of the lesion instead of necrotic tissue. Nerves and blood vessels should also be avoided. Finally, adequate specimens are also very important for making a pathological diagnosis. Wu *et al*. demonstrated that the diagnostic accuracy rate increased with the number of specimens obtained and with longer specimen length^12^. In their study, they suggested that the cumulative diagnostic yield reached a plateau at three specimens for bone lesions.

The complication rate of CT‐guided biopsy reported by other authors is 0% to 2%^5^, ^6^, ^8^, ^13^. Only minor complications are reported, such as hematomas and transient paresis. In our study, no complications were recorded.

There were several limitations in our study. It was a retrospective design instead of a randomized trial. In addition, in our study, the same patient did not receive two methods of biopsy. Therefore, it was not appropriate to directly use a receiver operating characteristic curve to compare the diagnostic accuracy between the two groups. Another limitation was that all the pathological diagnoses were not made by one pathologist. Finally, the sample size in our research was not large enough. Nevertheless, we believe that our study provides useful data for clinical work and that further study will be valuable.

In conclusion, CT‐guided core needle biopsy is an effective and minimally invasive procedure for diagnosing spinal lesions. It can be performed with high diagnostic accuracy and few complications. Therefore, it should be recommended as the first‐line biopsy for diagnosing spinal lesions.
